# Reduction in malaria prevalence and increase in malaria awareness in endemic districts of Bangladesh

**DOI:** 10.1186/s12936-016-1603-0

**Published:** 2016-11-11

**Authors:** Mohammad Shafiul Alam, Mohammad Moktadir Kabir, Mohammad Sharif Hossain, Shamsun Naher, Nur E. Naznin Ferdous, Wasif Ali Khan, Dinesh Mondal, Jahirul Karim, A. K. M. Shamsuzzaman, Be-Nazir Ahmed, Akramul Islam, Rashidul Haque

**Affiliations:** 1 icddr,b, 68 Shaheed Tajuddin Ahmed Sarani, Mohakhali, Dhaka, 1212, Bangladesh; 2 BRAC, 75 Mohakhali, Dhaka, Bangladesh; 3 NMCP Mohakhali, Dhaka, 1212 Bangladesh; 4 DGHS Mohakhali, Dhaka, 1212 Bangladesh

**Keywords:** Malaria, Prevalence, Survey, Awareness, Bangladesh

## Abstract

**Background:**

Malaria is endemic in 13 districts of Bangladesh. A baseline malaria prevalence survey across the endemic districts of Bangladesh was conducted in 2007, when the prevalence was reported around 39.7 per 1000 population. After two rounds of Global Fund to Fight AIDS, Tuberculosis and Malaria (GFATM)-funded intervention by the National Malaria Control Programme (NMCP) and a BRAC-led NGO consortium, a follow-up survey was conducted across the malaria-endemic districts of Bangladesh to measure the change in prevalence rate and in people’s knowledge of malaria.

**Methods:**

The survey was carried out from August to November 2013 in 70 *upazilas* (sub-districts) of 13 malaria-endemic districts of Bangladesh, following the same multi-stage cluster sampling design and the same number of households enrolled during the baseline prevalence survey in 2007, to collect 9750 randomly selected blood samples. For on-the-spot diagnosis of malaria, a rapid diagnostic test was used. The household head or eldest person available was interviewed using a pre-coded structured questionnaire to collect data on the knowledge and awareness of malaria in the household.

**Results:**

Based on a weighted calculation, the overall malaria prevalence was found to be 1.41 per 1000 population. The proportion of *Plasmodium falciparum* mono-infection was 77.78% while both *Plasmodium vivax* mono-infection and mixed infection of the two species were found to be 11.11%. Bandarban had the highest prevalence (6.67 per 1000 population). Knowledge of malaria signs, symptoms and mode of transmission were higher in the follow-up survey (97.26%) than the baseline survey. Use of bed nets for prevention of malaria was found to be high (90.15%) at respondent level. People’s knowledge of selected parameters increased significantly during the follow-up survey compared to the baseline survey conducted in 2007.

**Conclusions:**

A reduced prevalence rate of malaria and increased level of knowledge were observed in the present malaria prevalence survey in Bangladesh.

## Background

Globally, 96 countries and territories are endemic for malaria with an estimated 3.2 billion people at risk. The World Health Organization (WHO) estimated 214 million malaria cases worldwide annually (uncertainty range 149–303 million) with 438,000 deaths (uncertainty range 236,000–635,000) in 2015 [1]. Among the ten malaria-endemic countries of the WHO Southeast Asian region, Bangladesh is considered as hypo-endemic for malaria transmission where 90% of malaria is caused by *Plasmodium falciparum*. In Bangladesh, 13 districts (out of 64) are endemic for malaria, all of which border India and/or Myanmar and almost 80% of malaria cases are reported from three districts: Bandarban, Khagrachari and Rangamati (collectively called Chittagong Hill Tracts (CHT)) [2].

Bangladesh received two grants from the Global Fund to Fight AIDS, Tuberculosis and Malaria (GFATM), Round 6 (in 2007) to scale-up malaria control interventions, and another in 2009 (Round 9) for further expansion of intervention coverage [3]. The Government of Bangladesh and an NGO consortium led by BRAC has been implementing this GFATM-funded malaria control programme in 13 malaria-endemic districts since 2007. As a result, malaria prevalence has decreased by 65%, severe malaria decreased by 79%, and malaria-associated mortality decreased by 91% from 2008 to 2012 [4].

A baseline malaria prevalence survey was conducted across 13 endemic districts of Bangladesh in 2007 by the same group of investigators, when the prevalence was reported around 39.7 per 1000 population. The majority of cases was found to be caused by *P. falciparum* (90.18%) followed by *Plasmodium vivax* (5.29%) and mixed infection (4.53%) by these two species [5].

To measure the change in prevalence rate and in people’s knowledge of malaria from 2007, a follow-up malaria prevalence survey was conducted across the malaria-endemic areas of Bangladesh. The results of the follow-up survey and some comparisons with the baseline survey are presented here.

## Methods

### Study area

The survey was carried out in 70 *upazilas* (sub-districts) of 13 malaria-endemic districts of Bangladesh [5]. Epidemiologically, these districts can be divided into two groups: the CHT districts, where 80% of the country’s total malaria cases are reported every year, and the remainder (non-CHT). CHT is mostly hilly, forested and some parts are difficult to access [2]. Cox’s Bazar and Bandarban border Myanmar, Rangamati borders both India and Myanmar, while the rest of the malaria endemic districts share a border with India.

### Sample size and sampling

The same multi-stage cluster sampling design as was used in the 2007 baseline survey was followed to test 9750 individuals from an equal number of households [5]. Roughly, these prevalence surveys used a two-stage cluster design. City corporations and towns were excluded. For each of the 13 districts, all *mauzas* (the lowest administrative unit of Bangladesh, mostly bigger than a village and with a polygon boundary) were listed and 30 clusters were selected using a probability proportional to size (PPS) sampling procedure [6]. Information from the Bangladesh Bureau of Statistics was used to obtain the sampling frame for selection of the *mauzas* in each district. These *mauzas* were the primary sampling unit. All of the population, above one year old, irrespective of sex, religion or ethnicity, in a cluster were eligible to participate in the survey. Twenty-five households were selected using systematic random sampling from each cluster. As in the baseline survey [5], the household head or eldest person available was interviewed using a pre-coded, structured questionnaire to collect data on bed net use, knowledge and awareness to malaria by the household. For on-the-spot malaria diagnosis using rapid diagnostic test kit, one household member was chosen randomly from the available household members.

### Ethical approval

The protocol was reviewed and approved by the institutional research review committee and the ethical review committee of International Centre for Diarrhoeal Disease Research, Bangladesh (icddr,b). Written informed consent was obtained from all adult subjects, and assent was obtained from legal guardians in the case of minor subjects before starting of any study procedure.

### Field operation

The survey was conducted from August to November 2013. Households were selected systematically by the field team following a designated path using the ‘spin the bottle’ methodology [7] similar to the baseline survey [5].

All members of the household, including absentees, were listed. Only one individual (>one year) from a household was enrolled in this study using a simple randomization procedure. Written informed consent or assent was obtained before proceeding with the survey activities. Selected individuals were tested for malaria and information collected for any febrile illness in the previous 15 days.

### Malaria diagnosis

One team member drew four to five drops of blood from randomly selected participants to perform malaria test by a rapid diagnostic test (RDT), prepare a slide smear (both thick and thin) for microscopic examination, and spot blood onto a filter paper for further molecular diagnosis. For on-the-spot malaria diagnosis from blood sample of the selected individual, a FalciVax (Zephyr Biomedicals, India) RDT was used. The same RDT was used during the baseline prevalence survey [5]. This specific RDT can detect both *P. falciparum*-specific antigen (HRP-2) and *P. vivax*-specific antigen (pLDH) from the blood sample. This RDT has also shown acceptable performance (more than 90% in both sensitivity and specificity) in a field evaluation in Bangladesh [8].

The test results were recorded by the field team on the record sheet. Patients diagnosed as having malaria were provided treatment as per national guideline.

### Quality control

The day-to-day field activities of the teams were ‘fine-tuned’ by field researchers based in local offices. The investigators from central office at Dhaka made frequent field visits for on-spot checking of the quality of interviews and to provide assistance and guidance when needed. Whenever required, re-interviewing was carried out by the supervisors to secure reliable and valid data.

### Data management and analysis

All data from the field were recorded on pre-coded, paper-based forms, which were designed for direct data entry using a scanner. Field workers were trained to use the forms so that information could be read easily. ABBYY FlexiCapture 8.0 (ABBYY 3A, Otradnaya str. 2b/6, 127,273, Moscow, Russia) software for electronic data capturing and processing was used for data capturing. Data were then exported to a Microsoft Office Access 2011 database. The data files were maintained at the data centre in Dhaka and Bandarban. Images of the scanned data forms were maintained in digital format in case there was a need to inspect the primary data source. For data analysis ‘svy’ command of STATA (Version 11, StataCorp LP, TX, USA) was used. All results were weighted (weight = 1/probability of selection) to account for unequal probabilities of selection of clusters across district. Bivariate analysis was performed to determine the change regarding knowledge level of a respondent. A proportion test was used to detect any change in knowledge level between the baseline survey in 2007 and the present survey (2013). Differences between proportions were measured by one-tailed test in 5% (P < 0.05) level of significance.

## Results

### Malaria prevalence

Among the 13 malaria-endemic districts, the overall, unadjusted, malaria prevalence rate was 0.92 per 1000 population. The prevalence of *P. falciparum* mono-infection was 0.72 per 1000 population and 0.10 per 1000 population for *P. vivax* mono-infection, while for mixed infection with *P. falciparum* and *P. vivax* the prevalence was 0.10 per 1000 population. The proportion of *P. falciparum* mono-infection was 77.78% while for *P. vivax* mono-infection the proportion was 11.11% and for mixed infection the proportion was 11.11% in both cases. No significant difference in prevalence of malaria was detected with regard to the gender of study participants. Bandarban was the district with the highest prevalence (6.7 per 1000 population) followed by Cox’s Bazar (2.7 per 1000 population).

**Fig. 1:**
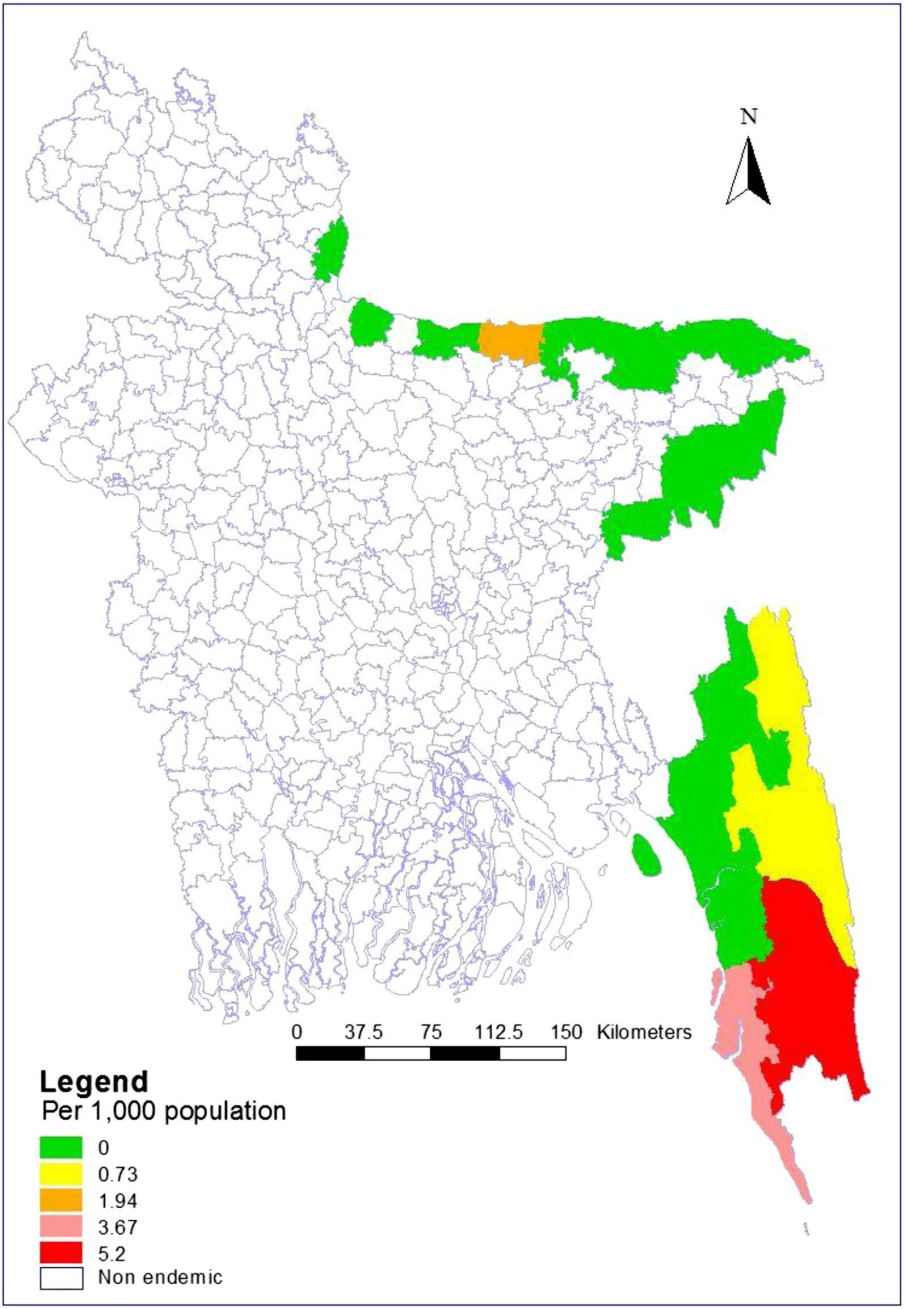
Prevalence (weighted) of malaria in endemic areas of Bangladesh in 2013

When considering the age group, children under five years of age were found to have a higher prevalence (7.37 per 1000) than the other groups. The average weighted malaria prevalence in CHT was 1.71 per 1000 population and in non-CHT districts 1.30 per 1000 population. Prevalence of malaria among symptomatic (febrile) individuals was 4.91 per 1000 population and 0.59 per 1000 population among asymptomatic individuals. Weighted prevalence of malaria according to infection type, gender, age group, area, and study participants are given in Table 1. Weighted prevalence of malaria in endemic areas of Bangladesh found in this survey is shown in Fig. 1.

**Table 1:** Prevalence of malaria (weighted) according to infection type, gender, age group, area, and study participants

	Prevalence (per thousand)			
	Any Plasmodium infection [95% CI]	Pf infection [95% CI]	Pv infection [95% CI]	Mixed (pf and pv) infection %[95% CI]
Overall	1.41 (0.56–3.50)	0.87 (0.31–2.44)	0.06 (0.01–0.41)	0.48 (0.07–3.40)
By Sex				
Male	2.38 (0.76–7.45)	2.38 (0.76–7.45)	0 (0,0)	0 (0,0)
Female	0.94 (0.21–4.28)	0.15 (0.02–1.04)	0.09 (0.01–0.61)	0.71 (0.10–5.03)
By age group (years)				
<4	7.37 (1.03–50.58)	7.37 (1.03–50.58)	0 (0)	0 (0)
5–14	0 (0)	0 (0)	0 (0)	0 (0)
15–49	1.76 (0.64–4.86)	1.00 (0.29–3.44)	0.08 (0.01–0.59)	0.68 (0.10–4.81)
≥50	0.34 (0.05–2.35)	0.34 (0.05–2.35)	0 (0)	0 (0)
By area				
CHT	1.71 (0.77–3.78)	1.48 (0.61–3.58)	0.23 (0.03–1.67)	0 (0)
Non CHT	1.30 (0.36–4.65)	0.66 (0.12–3.61)	0 (0)	0.64 (0.09–4.57)
Type of participants				
Symptomatic	4.91 (1.71–13.96)	2.37 (1.06–5.29)	0 (0)	2.53 (0.37–17.20)
Asymptomatic	0.59 (0.10–3.35)	0.52 (0.07–3.68)	0.07 (0.01–0.51)	0 (0)

### Knowledge of malaria

Malaria awareness-related responses are summarized in Table 2. People in CHT were found to have better malaria awareness than non-CHT areas. It was found that most households knew about malaria (97.76%). Most of the respondents (97.26%) also knew fever as the cardinal sign for malaria. The respondents’ awareness about malarial illness as well as its cause (mosquito bite) was very high (95.02%).

**Table 2:** Malaria awareness characteristics of the study population by study areas, N(%)

Characteristics	CHT	Non-CHT	All
Have heard about malaria			
Yes	2208 (98.13)	7324 (97.65)	9532 (97.76)
No	42 (1.87)	174 (2.32)	216 (2.22)
Don’t know	0	2 (0.03)	2 (0.02)
Symptoms of malaria			
Fever	2184 (97.07)	7299 (97.32)	9483 (97.26)
Fever with shivering	131 (5.82)	428 (5.71)	559 (5.73)
Don’t know	53 (2.36)	190 (2.53)	243 (2.49)
Others	43 (1.91)	5 (0.07)	48 (0.49)
Causes of malaria			
Mosquito bite	2153 (95.69)	7111 (94.81)	9264 (95.02)
Fly/insect bite	14 (0.62)	10 (0.13)	24 (0.25)
Not maintaining neat and cleanliness	62 (2.76)	81 (1.08)	143 (1.47)
Don’t know	81 (3.60)	351 (4.68)	432 (4.43)
Others	0	0	0
How to prevent malaria			
Preventing breeding of mosquito	585 (26.00)	488 (6.51)	1073 (11.01)
Using bed-net	1890 (84.00)	6900 (92.00)	8790 (90.15)
Using insecticide impregnated bed-net	1707 (75.87)	1885 (25.13)	3592 (36.84)
Using mosquito repellent/coil	400 (17.78)	2198 (29.31)	2598 (26.65)
Don’t know	49 (2.18)	226 (3.01)	275 (2.82)
Others	52 (2.31)	48 (0.64)	100 (1.03)
Place of seeking treatment			
Public hospital/health centre	1579 (70.18)	5115 (68.20)	6694 (68.66)
Private health centre	185 (8.22)	890 (11.87)	1075 (11.03)
Village doctors	280 (12.44)	890 (11.87)	1170 (12.00)
Drug store sales people	259 (11.51)	438 (5.84)	697 (7.15)
NGO	1722 (76.53)	3325 (44.33)	5047 (51.78)
Don’t know	40 (1.78)	651 (8.68)	691 (7.09)
Others	32 (1.42)	87 (1.16)	119 (1.22)
Have to spend money for malaria treatment?			
Yes	169 (7.51)	1256 (16.75)	1425 (14.62)
No	2070 (92.00)	5611 (74.81)	7681 (78.78)
Don’t know	11 (0.49)	633 (8.44)	644 (6.61)

Use of insecticide-treated bed nets (ITN) was found a preferred method for prevention (>90%). However, people in CHT (75.87%) thought ITN could be a better preventive method than non-CHT areas (25.13%).

People in CHT knew both public health facilities (70.18%) and NGO workers (76.53%) were among the preferred options for treatment. In non-CHT areas only 44.33% people knew about NGO workers, who provide malaria diagnosis and treatment. The majority of the respondents knew that treatment of malaria (78.78%) was provided free of cost (Table 2). All these better responses were found among respondents in CHT than in non-CHT (Table 2) but this difference was not found to be statistically significant.

People’s knowledge of symptoms of malaria as fever, transmission by mosquito bites, prevention by using bed net, ITN and mosquito-repellent coils, and preference to government hospital or partner NGO for treatment, was found to be increased significantly during follow-up survey in 2013 compared to the baseline survey in 2007 (Table 3).

**Table 3:** Comparison of people’s knowledge between baseline prevalence survey in 2007 and follow up survey in 2013

Variables	Malaria survey 2007 (µ_1_)	Malaria survey 2013 (µ_2_)	P value
	Proportion (CI)	Proportion (CI)	
How do you know that you have got malaria?			
Fever	0.943 (0.939–0.948)	0.990 (0.988–0.992)	<0.001
Others	0.135 (0.128–0.142)	0.071 (0.066–0.076)	<0.001
Why someone gets malaria?			
Mosquito bite	0.943 (0.938–0.948)	0.965 (0.961–0.969)	<0.001
Fly/insect bite	0.031 (0.027–0.034)	0.002 (0.001–0.003)	<0.001
Not maintaining cleanliness	0.052 (0.048–0.057)	0.015 (0.012–0.017)	<0.001
Others	0.043 (0.039–0.047)	0.030 (0.026–0.033)	<0.001
How malaria could be prevented?			
Preventing breeding	0.161 (0.154–0.168)	0.112 (0.106–0.119)	<0.001
Using bed net	0.854 (0.847–0.862)	0.914 (0.908–0.920)	<0.001
Using insecticide bed-net	0.017 (0.014–0.020)	0.376 (0.366–0.386)	<0.001
Coil	0.187 (0.180–0.195)	0.269 (0.260–0.278)	<0.001
Others	0.108 (0.101–0.114)	0.025 (0.022–0.028)	<0.001
Where you can get this treatment?			
Govt. hospital	0.677 (0.668–0.686)	0.696 (0.687–0.705)	<0.001
Private health centre	0.206 (0.198–0.214)	0.112 (0.106–0.118)	<0.001
Village doctors	0.387 (0.377–0.397)	0.121 (0.115–0.128)	<0.001
Drug stores	0.294 (0.285–0.303)	0.072 (0.067–0.077)	<0.001
NGO	0.245 (0.237–0.254)	0.548 (0.538–0.558)	<0.001

## Discussion

Bangladesh is among the six Southeast Asian countries to report at least 75% decrease in malaria incidence of confirmed cases due to delivery of vector control interventions (ITNs or indoor residual spraying (IRS)) to protect more than 60% of its population at high risk between 2000 and 2014 [1]. Following the last few years of GFATM-supported control initiatives, Bangladesh has made significant progress in malaria control. The study documents the reduction of the prevalence rate of malaria infection and increased knowledge of malaria by the endemic population of Bangladesh compared to the baseline survey conducted in 2007 [5]. High coverage of and increased use of ITN, RDTs and anti-malarial treatment with artemisinin combination therapy (artemether–lumefantrine), and the high number of community health workers and health facilities, has contributed significantly to this achievement [4]. For the same reason low malaria prevalence has been observed in many countries, such as reported in Eritrea [9].

As there is a possibility of the presence of asymptomatic malaria cases, as seen in previous observations in Bandarban district of CHT [10, 11], it could not be overruled that a PCR-based molecular diagnosis would be useful to detect asymptomatic individuals within the population surveyed in the study. In Cambodia by PCR, overall malaria prevalence was found to be almost 2.5-fold higher than was estimated by RDT [12]. Thus, the major constraint of the present report is the absence of supporting evidence from molecular tests such as PCR. On the other hand, there is a rare possibility of low positivity in RDT due to intra-specific variation in gene encoding the HRP(Histidine-rich protein) [13] or deletion of the gene [14] among *P. falciparum* isolates. There is documentation of low positivity in RDT recently in the Sylhet area of Bangladesh due to deletion of the gene (unpublished data); the sequence is reported to the Genbank (accession number: KH388531).

Overall, knowledge of malaria transmission has improved since the baseline survey [15] as evident from the present survey. It is understandable that in malaria-endemic communities, with ongoing control activities, increased knowledge should prevail. This was found to be the case in this survey as well as in Swaziland [16], Ethiopia [17], Eritrea [9], and Nigeria [18]. Knowledge on the use of ITN as a preventive measure against mosquito bites was found to be significantly higher among respondents in this follow-up study compared to the baseline survey (Table 3) [15]. This level of knowledge on preventive use of ITN has also been observed in Colombia [19], Nepal [20], Eritrea [9], and Ghana [21] while a lower level of knowledge was observed in India [22], Iran [23] and Turkey [24].

It has been observed that artemisinin-based combination therapy remains highly effective for the treatment of malaria caused by *P. falciparum* in Bangladesh [25]. However, the greatest threat to the malaria control programme in Bangladesh remains the increasing artemisinin resistance emerging in Southeast Asia. There, two independent foci of resistance based on K13-propeller mutations in *P. falciparum* have been identified, one of which includes Cambodia, Vietnam and Laos and the other includes western Thailand, Myanmar and China [26]. Moreover, it has been reported that the K13-propeller mutations in *P. falciparum* reached high prevalence in Myanmar, next to the northwestern border with India [27]. Such emergence of artemisinin resistance to *P. falciparum* strains in neighbouring India and Myanmar could be transmitted through malaria vectors active in remote border areas of Bangladesh [28, 29].

Reported resistance to pyrethroids by malaria vectors can be seen as another important obstacle to malaria control activities in future. Bangladesh, along with its neighbours India and Myanmar, has reported malaria vector resistance to pyrethroids, the main insecticide group used in ITN by control programmes [1].

## Conclusions

Reduction of malaria prevalence and an increased level of awareness of malaria has a positive health impact through active collaboration between NMCP, GoB (Government of Bangladesh) and NGO partners in Bangladesh. Involvement of community participation is crucial, not only to sustain this achievement but for its further enhancement. Efforts should be made to continue ongoing malaria control activities with the aim of eliminating malaria from endemic areas.

## Abbreviations

CHT: Chittagong Hill Tract
GFATM: Global Funds to Fight AIDS, Tuberculosis and Malaria
GoB: Government of Bangladesh
HRP: histidine-rich protein
icddr,b: International Centre for Diarrhoeal Disease Research, Bangladesh
ITN: insecticide-treated net
NGO: non-governmental organization
NMCP: National Malaria Control Programme
pLDH: *Plasmodium* lactate dehydrogenase
PPS: probability proportional to size
RDT: rapid diagnostic test
